# Prognostic Significance of N-Glycolyl GM3 Ganglioside Expression in Non-Small Cell Lung Carcinoma Patients: New Evidences

**DOI:** 10.1155/2015/132326

**Published:** 2015-11-08

**Authors:** Rancés Blanco, Elizabeth Domínguez, Orlando Morales, Damián Blanco, Darel Martínez, Charles E. Rengifo, Carmen Viada, Mercedes Cedeño, Enrique Rengifo, Adriana Carr

**Affiliations:** ^1^Laboratory of Recognition and Biological Activity Assays, Center of Molecular Immunology, 216 Street and 15th Avenue, Atabey, Playa, P.O. Box 16040, 11600 Havana, Cuba; ^2^Laboratory of Biochemistry, Department of Quality Control, Center of Molecular Immunology, 216 Street and 15th Avenue, Atabey, Playa, P.O. Box 16040, 11600 Havana, Cuba; ^3^Process Development Direction, Center of Molecular Immunology, 216 Street and 15th Avenue, Atabey, Playa, P.O. Box 16040, 11600 Havana, Cuba; ^4^Department of Cell Biology and Tissues Banking, National Institute of Oncology and Radiobiology, 29 and F Street, Vedado, Plaza de la Revolución, 10400 Havana, Cuba; ^5^Tumor Immunology Direction, Center of Molecular Immunology, 216 Street and 15th Avenue, Atabey, Playa, P.O. Box 16040, 11600 Havana, Cuba; ^6^Department of Pathology, Manuel Fajardo General Hospital, Zapata and D Street, Vedado, Plaza de la Revolución, 10400 Havana, Cuba; ^7^Clinical Trials Direction, Center of Molecular Immunology, 216 Street and 15th Avenue, Atabey, Playa, P.O. Box 16040, 11600 Havana, Cuba; ^8^Research and Development Direction, Center of Molecular Immunology, 216 Street and 15th Avenue, Atabey, Playa, P.O. Box 16040, 11600 Havana, Cuba

## Abstract

The prognostic role of N-glycolyl GM3 ganglioside (NeuGcGM3) expression in non-small cell lung carcinoma (NSCLC) still remains controversial. In this study, the NeuGcGM3 expression was reevaluated using an increased number of NSCLC cases and the 14F7 Mab (a highly specific IgG1 raised against NeuGcGM3). An immunohistochemical score integrating the percentage of 14F7-positive cells and the intensity of reaction was applied to reassess the relationship between NeuGcGM3 expression, some clinicopathological features, and the overall survival (OS) of NSCLC patients. The double and the triple expression of NeuGcGM3 with the epidermal growth factor receptor (EGFR) and/or its ligand, the epidermal growth factor (EGF), were also evaluated. NeuGcGM3 expression correlates with both S-Phase fraction (*p* = 0.006) and proliferation index (*p* = 0.000). Additionally, NeuGcGM3 expression was associated with a poor OS of patients in both univariate (*p* = 0.020) and multivariate (*p* = 0.010) analysis. Moreover, the double and/or the triple positivity of tumors to NeuGcGM3, EGFR, and/or EGF permitted us to identify phenotypes of NSCLC with a more aggressive biological behavior. Our results are in agreement with the negative prognostic significance of NeuGcGM3 expression in NSCLC patients. However, standardization of techniques to determine the expression of NeuGcGM3 in NSCLC as well as the implementation of a universal scoring system is recommended.

## 1. Introduction

Lung cancer is one of the most frequent cancers in the world and usually has a very poor prognosis. There are two main forms of the disease but non-small cell lung cancer (NSCLC) represents about 80–85% of these tumors [1, 2]. Consequently, numerous studies are currently focusing on the selection of newer biological and molecular prognostic factors as a potential complement of TNM (tumor, node, metastasis) staging system [3–5]. In line with this, unusual glycosylated gangliosides have been identified by immunohistochemistry (IHC) in NSCLC, also becoming attractive targets for immunotherapy [6, 7].

The aberrant expression of N-glycolyl GM3 ganglioside (NeuGc*α*2-3Gal*β*1-4Glc*β*-Cer) (NeuGcGM3) in NSCLC, by mean of 14F7 Mab reactivity, using frozen [8] and formalin-fixed and paraffin-embedded (FFPE) [9, 10] tissues, was previously demonstrated. This Mab is a highly specific IgG1 raised against NeuGcGM3 that does not cross-react with other NeuGc-containing gangliosides [11]. In addition, the expression of NeuGcGM3 was also evidenced by immunohistochemistry and TLC-immunostaining in FFPE samples with GMR8 Mab [7]. GMR8 Mab is an IgM that reacts with an epitope shared by more than one of the NeuGc-containing gangliosides [12]. In line with this, at least another NeuGc-containing ganglioside, GD1a (NeuGc), was recognized by GMR8 Mab in NSCLC tissues, although NeuGcGM3 was the predominant ganglioside identified in these samples [7].

Additionally, the relevance of NeuGcGM3 in cancer progression and its capability of modulating CD4 expression on T cells have been published [13, 14]. Recently, the expression of NeuGcGM3, as shown as 14F7 Mab staining, has been associated with more aggressive disease in colon adenocarcinoma [15]. In NSCLC patients, the expression of NeuGcGM3 inhibits dendritic cells (DCs) differentiation, maturation, and migration leading to tumor-induced DCs suppression [9]. However, at present, the prognostic role of NeuGcGM3 expression in NSCLC still remains controversial [7, 9, 10].

For this reason, in this study the prognostic value of the 14F7 Mab reactivity was reevaluated using an increased number of Cuban NSCLC patients as well as an immunohistochemical score, which integrate the percentage of positive cells and the intensity of reaction. Additionally, the impact of the simultaneous expression of NeuGcGM3, epidermal growth factor receptor (EGFR), and/or epidermal growth factor (EGF) was assessed in the overall survival of these patients. A study of the antigen recognized by 14F7 Mab in FFPE tissues was also included.

## 2. Materials and Methods

### 2.1. Monoclonal Antibodies

We used the 14F7, a murine IgG1 highly specific against the N-glycosylated variant of GM3 ganglioside [11], the ior egf/r3 (anti-human EGFR) [16], and the CB-EGF1 (anti-human EGF) [17]. 14F7 and ior egf/r3 Mabs were produced by the Center for Molecular Immunology (Havana, Cuba), while CB-EGF1 was kindly provided by the Center for Genetic Engineering and Biotechnology (Havana, Cuba). Additionally, the anti-human EGFR Mab (Dako EGFR pharmDx kit, K1494), the negative control included in this kit, the P3 Mab, an IgM that recognize NeuGcGM3, and other NeuGc-containing gangliosides as well as sulfated glycolipids [6] were employed.

### 2.2. Tissue Specimens

A total of 90 formalin-fixed and paraffin-embedded (FFPE) tissues with diagnosis of NSCLC were received from the pathology department of both Hermanos Ameijeiras General Hospital and the National Institute of Oncology and Radiobiology, after approved consent by the institutional ethical committees. Specimens from patients with stages I–IIIA were surgically resected by means of wedge biopsy, lobectomy, or pneumonectomy before the standard radio-chemotherapeutic regimens, while samples from patients with stages IIIB–IV were obtained during the clinical autopsy.

### 2.3. Lipid Extraction

Paraffin blocks were melted by heating in a 70°C oven for 4 h. The tissue samples were maintained in xylene for 1 h at 70°C and then at room temperature (3 × 1 h). The tissues were rehydrated in graded ethanol series (100%, 100%, 90%, and 70%) for 1 h each. Finally, they were washed in tap and distilled water for 30 and 15 minutes, respectively. For the lipid extraction, the method as it was previously described by Bligh and Dyer (1959) with minor modifications was used [18]. Briefly, one of two fragments of NSCLC samples (1-2 g) was mechanically homogenized. Then, 3.75 mL of a mixture chloroform/methanol (1/2) was added and vortexed for 30 minutes. Afterward, 1.25 mL of chloroform and 1.25 mL of distilled water were added and the preparation was mixed during 1 minute in each step. The samples were centrifuged, the upper phase was discarded, and finally the lower phase was collected with a Pasteur pipette. After evaporation, the lipid extract (lower phase) was redissolved in 20 *μ*L of chloroform/methanol (2/1).

### 2.4. Thin-Layer Chromatography

Glycolipids extracted from paraffin blocks were immunostained on a high-performance thin-layer chromatography (HPTLC) aluminium plates (Merck-Millipore, 105553) to determine the ganglioside fraction. The solvent system used for developing chromatograms was chloroform/methanol/0.25% ammonia (5 : 4 : 1, v/v/v). Afterward, the plates were air dried and carbohydrates were visualized with orcinol stain. For immunostaining, after chromatography, plates were soaked for 75 seconds on hexane (Sigma-Aldrich, 296090) containing 0.1% poly (isobutyl methacrylate) (Sigma-Aldrich, 445754). The plates were air dried, blocked with phosphate buffer saline (PBS) (Capricorn, PBS-1A) containing 1% bovine serum albumin (BSA) (Sigma-Aldrich, A2153) for 30 minutes, and incubated with 20 *μ*g/mL of 14F7 Mab for 3 hours at room temperature. Afterward, the plates were washed with PBS/BSA 1% and incubated with peroxidase-conjugated goat anti-mouse IgG antibody (Sigma-Aldrich, A4416). The reaction was developed with 3,3-diaminobenzidine (Sigma-Aldrich, D800-1).

### 2.5. Mass Spectrometry

The gangliosides fractions extracted from FFPE tissues were analyzed by matrix-assisted laser desorption/ionization-time of flight (MALDI TOF) mass spectrometry. MALDI mass spectra were obtained using an AXIMA Performance MALDI TOF/TOF mass spectrometer (Shimadzu Biotech) with a 337 nm N_2_ laser using delayed extraction, in positive reflectron mode. The glycolipid extracts were resuspended in 10 *μ*L of 50% methanol, from which 1 *μ*L was mixed with an equal volume of a saturated solution of 2,5-dihydroxybenzoic acid (DHB) (Sigma-Aldrich, 85707) in 50% acetonitrile (Sigma-Aldrich, 34851) on top of the MALDI target plate. Samples were allowed to dry off at room temperature and were recrystallized through the addition of 1 *μ*L of ethanol. Two hundred laser shots were accumulated for each mass spectrum, with a 50 Hz laser repetition rate. In this analysis, signals between* m/z* 1.000 and 2.000 were collected. The data was processed using MALDI-MS software from Axima Biotech Launch pad software pack.

The spectra were externally calibrated against ProteoMassTM peptide MALDI-MS calibration kit (Sigma-Aldrich, MSCAL2-1KT). The peaks corresponding to NeuAcGM3 and NeuGcGM3 were confirmed by comparison with purified samples of both gangliosides.

### 2.6. Immunohistochemical Staining

Five-micrometer serial sections from each block were obtained in a micrometer (Leitz, 1512) and they were mounted on plus slides (Dako, S2024). All sections were attached to the slide by heating in a 60°C oven for 1 h. Afterward, the slides were dewaxed in xylene and rehydrated in graded ethanol series in the usual way. The samples were maintained in tap water until they were stained.

The immunolocalization of NeuGcGM3 ganglioside was performed as it was previously described in [10] with some modifications. Briefly, the slides were incubated with 14F7 Mab in a humid chamber for 1 h at room temperature followed by the labeled streptavidin biotin (LSAB) two steps' system (Dako, K0690) both for 30 minutes at room temperature. The enzymatic activity was visualized with 3,3-diaminobenzidine (DAB) substrate chromogenic solution (Dako, K3465) and the tissues were counterstained with Mayer's Hematoxylin (Dako, S2020). Concerning the evaluation of both EGFR and EGF tissue antigens, the procedure as it was previously described in [19] was used.

### 2.7. Enzymatic Tissue Treatments

In essence, tissue treatments were performed as described by Kotani and Tai [20] with some variations to formalin-fixed and paraffin-embedded samples. After routine dewaxing and rehydration, tissue sections were treated with 4 U/mL of Neuraminidase* Clostridium perfringens *(Sigma-Aldrich, N2876) in PBS (Capricorn, PBS-1A) for 2 hours at 37°C. The enzyme from* Clostridium perfringens* cleaves terminal sialic acid residues which are *α*-2,3-, *α*-2,6-, or *α*-2,8-linked to Gal, GlcNac, GalNAc, AcNeu, GlcNeu, oligosaccharides, glycolipids, or glycoproteins. The relative rate of cleavage decreases in the order: *α*-2-3 > *α*-2-6, and *α*-2-8. As positive control, the antibody specific to the cell membrane glycoprotein EGFR (Dako, K1494) as well as the P3 Mab [6] was used. Slides incubated with the negative reactive (Dako, K1494) served as negative control.

### 2.8. Immunohistochemical Evaluation

The tissue expression of the NeuGcGM3, EGFR, and EGF in both cell membrane and cytoplasm of epithelial malignant cells was evaluated for percentage of positive cells and intensity of reaction. The most representative regions of each section were selected and the percentage of tumor cells showing immunostaining in them was estimated (0–100%) using the 10x objective lens. The intensity of reaction of each sample was judged as negative (0), weak (1), moderate (2), and strong (3). The final results were considered according to two observers' agreement (Charles E. Rengifo and Rancés Blanco).

### 2.9. Generation of Integrated IHC Scores

The percentage of positive cells and the intensity of reaction were multiplied for each specimen, resulting in a score ranging from 0 to 300. These scores ranges were grouped as follows: 0 (0), 1 (1 to 100), 2 (101–200), and 3 (>200) to generate an integrated score (*H*-score). Subsequently, the *H*-scores were grouped in this manner: low expression (scores < 150) and high expression (scores ≥ 150) to create the final score (*F*-score).

### 2.10. DNA Content, S and G_2_/M Phases Measurements

The flow cytometry methodology using nuclei isolated from formalin-fixed and paraffin-embedded tissues was used as it was previously described in [5]. The percentage of cells in S and G_2_/M phases of cell cycle was calculated for each sample based on DNA histograms. The S-Phase fraction (SPF) values were divided into two groups: a low-SPF group defined as having SPF less than 20% of proliferating cells and a high-SPF group with SPF greater than 20%. The proliferation index was calculated as the sum of cells in S and G_2_/M phases of cell cycle and was scored as it was previously described for SPF.

### 2.11. Statistical Analysis

The relationships between 14F7Mab immunoreactivity and clinicopathological variables were analyzed using the Chi-square test. Correlations were assessed by Spearman ranks correlation coefficients. Survival distribution was estimated by the Kaplan-Meier method and compared with the log-rank test. For multivariate analysis, the Cox regression model was used to identify independent prognostic factors for overall survival (OS). The model included all variables and the backward stepwise elimination as the algorithm of selection was applied. OS was measured from the date of surgery to death for any cause or last follow-up (5 years) and was calculated for all patients. A *p* value < 0.05 was considered statistically significant. Statistical analysis was carried out using SPSS (version 15.0; SPSS Inc., Chicago, IL).

## 3. Results

### 3.1. Patient Description and Clinicopathological Features

Table 1 shows a summary of patient characteristics and some pathological features. The gender ratio was close to 2 : 1 in favor of males, with a mean age of 57.4 ± 10.6 years. In general, the overall rate of NSCLC patients was 67.8% (61/90) and the median overall survival of this population at 5 years was 46.2 months (ranging from 0.7 to 67.1).

### 3.2. NeuGcGM3 Detection by Immunohistochemical and Biochemical Methods

**Figure 1:**
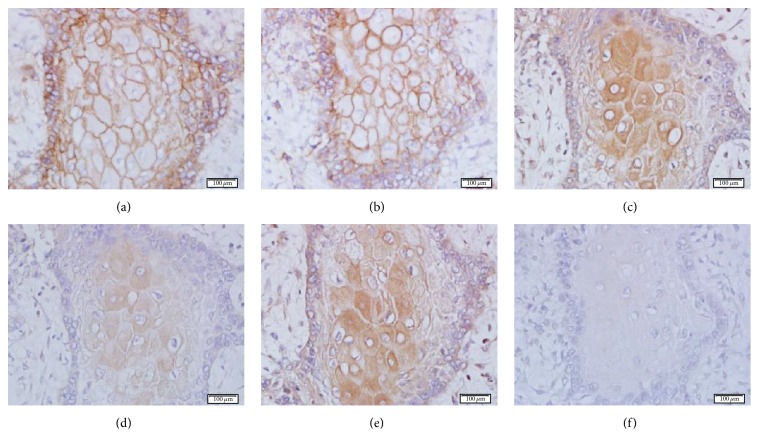
Microphotographs of formalin-fixed and paraffin-embedded NSCLC sections. Intense tissue reactivity of anti-EGFR (a), P3 (c), and 14F7 (e) Mabs without Neuraminidase (*Clostridium perfringens*) treatment. The expression of EGFR was not altered after enzymatic treatment (a), while the reactivity of P3 Mab was reduced but not completely eliminated (d) (brown color). Observe that the staining with 14F7 Mab was significantly removed after sialidase digestion (f) (brown color). Counterstaining with Mayer's Hematoxylin (blue color). White bar = 100 *μ*m.

Firstly, the specificity of the 14F7 Mab immunoreaction was confirmed with sialidase (*Clostridium perfringens*) treatment of NSCLC sections. The enzymatic digestion of tissues did not alter the level of EGFR expression, while the reactivity of P3 Mab was only reduced with this treatment (Figure 1). The staining with 14F7 Mab was significantly removed after sialidase digestion of tissue sections. No reaction was evidenced in NSCLC samples incubated with the negative control.

**Figure 2:**
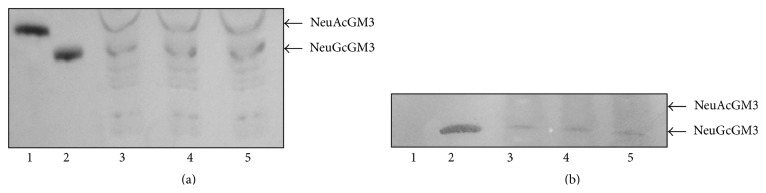
HP-TLC of glycolipids fraction isolated from FFPE samples of NSCLC. (a) Chemical staining with orcinol. 1 and 2: purified controls of NeuAcGM3 and NeuGcGM3, respectively. 3–5: glycolipids fraction extracted from NSCLC samples. Note the expression of both NeuAcGM3 and NeuGcGM3 gangliosides. (b) TLC-immunostaining using 14F7 Mab. Observe that the 14F7 Mab only reacted with the NeuGc-containing variant of GM3 ganglioside.

**Figure 3:**
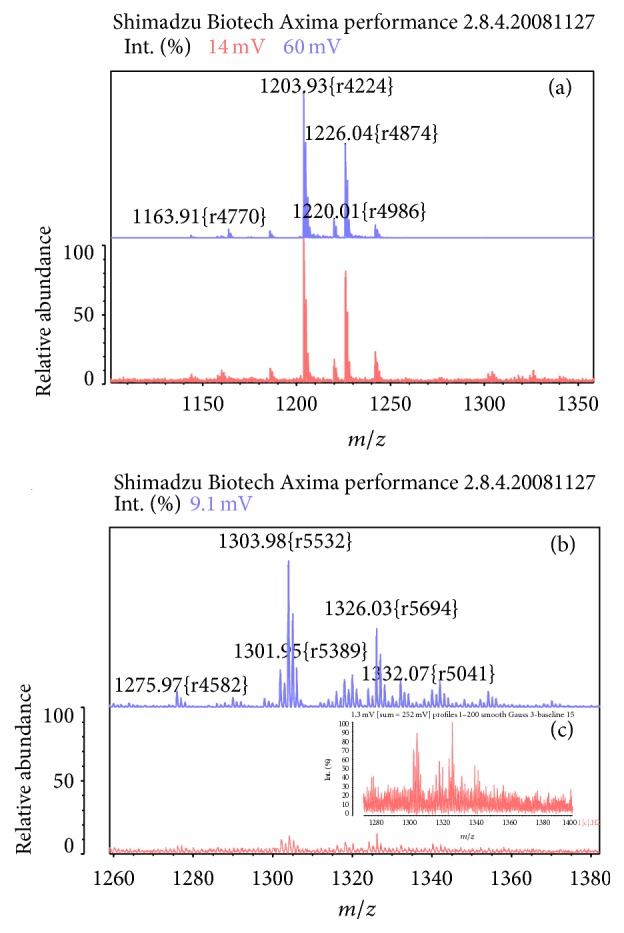
MALDI/TOF mass spectra of gangliosides extracted from NSCLC tissues. (a) and (b) (upper): purified controls of NeuAcGM3 and NeuGcGM3 gangliosides, respectively. Lower parts of panels (a) and (b) correspond to extracted fraction of NeuAcGM3 and NeuGcGM3 after the routine histological procedures. (c) Amplification of the lower part of panel (b).

TLC-immunostaining of gangliosides fraction extracted from 3 different histological subtypes of NSCLC (epidermoid carcinoma, adenocarcinoma, and large cell carcinoma) that were positive for NeuGcGM3 in the immunohistochemistry study was performed. Both NeuAcGM3 and NeuGcGM3 gangliosides were evidenced in all NSCLC samples (Figure 2(a)). However, only NeuGcGM3 was recognized by 14F7 Mab, while no reaction was evidenced with NeuAcGM3 (Figure 2(b)). The expression of these gangliosides after formalin fixation and routine histological procedures was also confirmed by mass spectrometry (Figure 3). NeuAcGM3 was the major ganglioside detected in NSCLC samples using this technology, although a little amount of NeuGcGM3 was also evidenced.

### 3.3. Immunohistochemical Detection of NeuGcGM3

The tissue expression of NeuGcGM3 was observed in 84/90 (93.3%) cases, with variable intensity of reaction and percentage of positive cells. Most specimens had a moderate to intense staining (76.7%) and more than 50% of positive cells (64.4%) as shown in Table 2. A significant correlation was found between percentage of positive tumor cells and staining intensity (*p* < 0.000; Spearman's correlation coefficient = 0.725). According to the *F*-score, 58.9% of cases showed high level of NeuGcGM3 expression. The reactivity of 14F7 Mab was observed on both plasmatic membrane and cytoplasm of malignant cells with a homogeneous and finely granular pattern of staining as it was previously described in [8, 10].

### 3.4. Association of NeuGcGM3 Expression with Clinicopathological Features

The relation of 14F7 Mab immunostaining with clinicopathological variables is shown in Table 3. No significant differences were observed with gender, age, tumor size, occurrence of lymph node and other sites metastasis, stage, disease recurrence, grade of differentiation, and ploidy. However, a statistically significant difference was obtained when the reactivity of 14F7 Mab was compared in function of the histological subtype of tumors (*p* = 0.008; Chi-square test). Additionally, the level of NeuGcGM3 expression showed statistical correlation with both S-Phase fraction and index of cell proliferation (*p* = 0.006 and *p* = 0.000; Fisher exact test, resp.) (Table 4). When cases were analyzed independently, according to the intensity of reaction or the percentage of positive cells (data not shown), no significant associations with clinicopathological features were obtained. Nevertheless, higher levels of 14F7 Mab positive cells correlated with both S-Phase fraction and index of cell proliferation (*p* = 0.028 and *p* = 0.000; Fisher exact test, resp.), while for the intensity of reaction only a significant association (*p* = 0.037; Fisher exact test) was obtained with the proliferation index.

### 3.5. Survival Analysis

**Figure 4:**
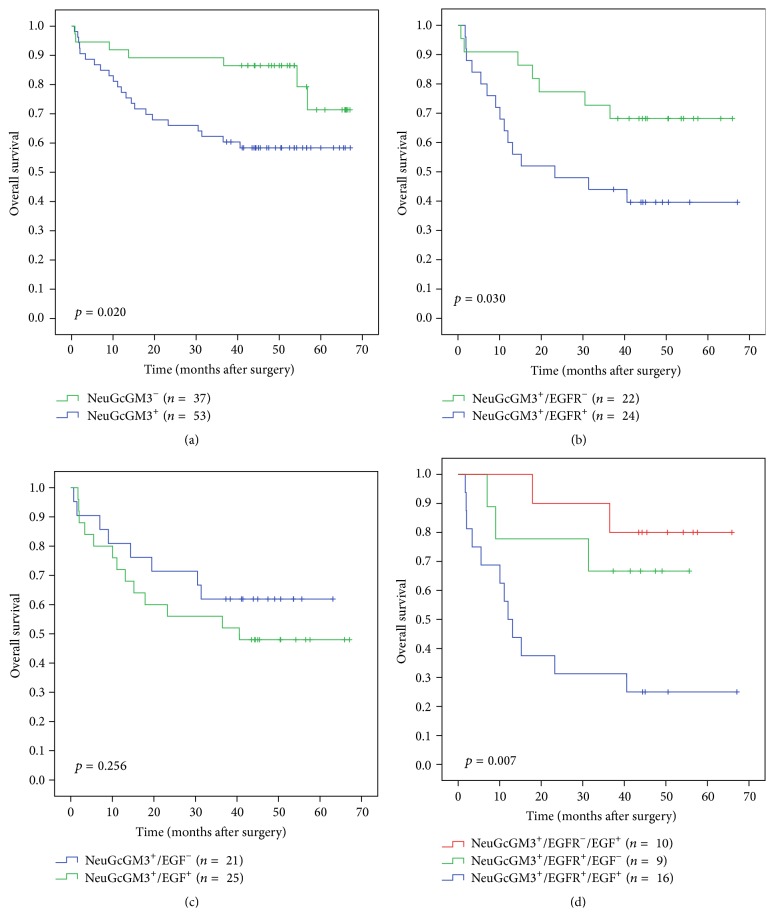
Kaplan-Meier curves for overall survival of NSCLC patients. (a) Expression of NeuGcGM3. (b) Dual expression of NeuGcGM3 and EGFR. (c) Double expression of NeuGcGM3 and EGF. (d) Triple expression of NeuGcGM3, EGFR, and EGF. Statistical analysis by log-rank test.

The results of univariate and multivariate survival analyses at 5 years are summarized in Table 5. Univariate analysis showed that lymph node metastasis (*p* = 0.000), other sites metastasis (*p* = 0.000), stage (*p* = 0.000), disease recurrence (*p* = 0.000), and the expression of NeuGcGM3 (*p* = 0.020) were significant prognostic factors for OS. Patients with high level of NeuGcGM3 expression had significantly impaired OS compared to those with low level by mean of the *F*-score (58.5% versus 81.1%; *p* = 0.001; Fisher's exact test). Five-year survival probabilities in NSCLC patients with higher levels of NeuGcGM3 expression were 3.4 times lower than those with reduced levels of this ganglioside. Kaplan-Meier curves according to 14F7 reactivity are represented in Figure 4(a). Among the studied variables, gender (*p* = 0.047), tumor size (*p* = 0.003), other sites metastasis (*p* = 0.029), disease recurrence (*p* = 0.001), and NeuGcGM3 expression (*p* = 0.010) were independent prognostic factors on multivariate analysis.

No association was obtained when the intensity of reaction or the percentages of positive cells were analyzed (*p* = 0.134 and *p* = 0.113; log-rank test, resp.).

### 3.6. Double and/or Triple Expression of NeuGcGM3, EGFR, and EGF Molecules

Higher levels of EGFR or EGF expression were observed in 58/80 (72.5%) and 67/80 (83.7%) of NSCLC samples, respectively. However, no correlation between the single expression of EGFR or EGF with the overall survival of NSCLC patients was obtained (data not shown). In addition, the dual expression of these molecules was only evidenced in 27/80 (33.7%) of cases. The patients with tumor displaying the phenotype EGFR^+^/EGF^+^ had a significantly impaired OS compared to those with EGFR^+^/EGF^−^ (48.1% versus 84.2%; *p* < 0.000; Fisher's exact test) (Table 6).

The double expression of NeuGcGM3 with EGFR or EGF was observed in 52/80 (65.0%) and 62/80 (77.5%) of cases, respectively. Patients with tumor NeuGcGM3^+^/EGFR^+^ had a significantly poorer OS than those with NeuGcGM3^+^/EGFR^−^ phenotype (40.0% versus 68.2%; *p* < 0.000; Fisher's exact test) (Figure 4(b)). But no association with the OS of patients was evidenced when NeuGcGM3^+^/EGF^+^ and NeuGcGM3^+^/EGF^−^ phenotypes of NSCLC were compared (Figure 4(c)).

**Figure 5:**
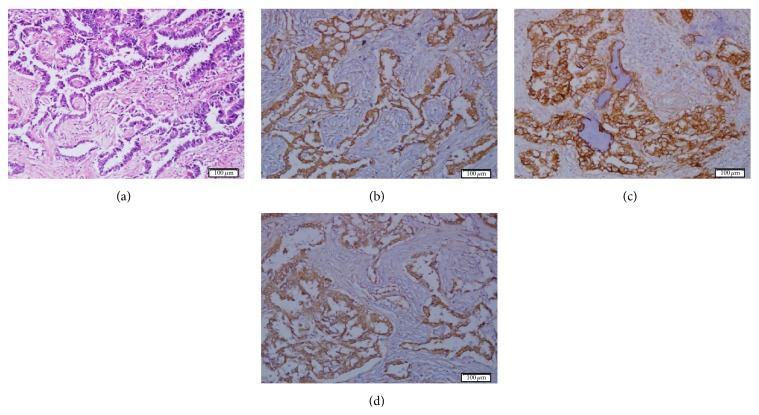
Triple expression of NeuGcGM3, EGFR, and EGF in lung adenocarcinoma. (a) Hematoxylin and eosin staining. ((b)–(d)) High levels of NeuGcGM3, EGFR, and EGF expression, respectively (brown color). Counterstaining with Mayer's Hematoxylin (blue color). White bar = 100 *μ*m.

Finally, the triple expression of NeuGcGM3, EGFR, and EGF was only detected in 16/80 (20.0%) of tumors (Figure 5). These patients displayed significantly reduced 5-year OS rates (*p* = 0.007; log-rank test) compared to those with NeuGcGM3^+^/EGFR^+^/EGF^−^ or NeuGcGM3^+^/EGFR^−^/EGF^+^ (25.0% versus 66.7% versus 80.0%; *p* < 0.000, Chi-square test) (Figure 4(d)), respectively.

## 4. Discussion

Despite recent advances in the diagnosis and treatment, lung cancer still remains the leading cause of death from cancer worldwide and one of the 10 leading causes of death from all causes [21]. Usually patients with NSCLC have a poor prognosis because most of them present with advanced or metastatic disease at the time of diagnosis [22]. Even after early diagnosis and surgical resection with curative intent, in stages I–III NSCLC patients, recurrent disease or distant metastasis results in a 5-year survival rate of <50% [23–25]. It has been estimated that only 10–15% of NSCLC patients will ultimately be cured [22]. In this sense, a number of independent prognostic factors for survival in NSCLC patients have been evaluated, including disease stage, performance status, gender, age, histology, haemoglobin level, some primary tumor characteristics such as tumor size or local extension, and mediastinal neoplastic infiltration [22].

In this study, we showed the prognostic significance of gender, tumor size, the occurrence of lymph node and other sites metastasis, stage, and disease recurrence in NSCLC patients. However, only the existence of other sites metastasis and the disease recurrence were identified as prognostic factor in both univariate and multivariate analyses. In previous reports, TNM (tumor, node, metastasis) staging system [26], disease stage, and performance status at time of diagnosis have been considered among most noticeable prognostic factors in these patients [22]. Nevertheless, the pathology-based TNM stage classification has been considered to provide imprecise information about the survival rates [27]. Since outcomes can be different even among patients with the same disease stage, numerous studies are currently focusing on the evaluation of other biological and molecular prognostic factors as a potential complement of TNM staging system [24, 25, 28].

GM3 is the major glycosphingolipid expressed in both tumor and nontumor tissues from human lung cancer patients [29]. In non-small cell lung cancer (NSCLC), an increased expression of GM3 and GM3 synthase (sialyltransferase-I or SAT-I) mRNA has been detected. However, the cDNA sequences of the cytidine monophospho-N-acetylneuraminic acid hydroxylase (CMAH) suggest an inactivating mutation, similar to that in normal tissues [7]. In this sense, these results suggest the existence of an alternative pathway for NeuGc-containing gangliosides, different from the normal pathway that is mediated by CMAH enzyme activity [30]. In line with this, Yin et al. reported a hypoxic culture induced NeuGc-containing ganglioside expression on human cells as a possible candidate for the alternative pathway because a hypoxic culture markedly induced mRNA for the sialic acid transporter, sialin [31].

In this study, the expression of NeuGcGM3 in NSCLC samples after the routine histopathological procedures was confirmed using both immunohistochemistry after sialidase digestion and TLC-immunostaining with 14F7 Mab. However, GM3 was the major ganglioside detected in this kind of samples, similar to previous report [7]. The tissue expression of NeuGcGM3 was confirmed by mean of mass spectrometry of lipid fraction extracted from FFPE samples. NeuGcGM3 is not completely extracted from NSCLC tissues after formaldehyde fixation and the subsequent histological procedures [8]. In a previous work, our group published the NeuGcGM3 ganglioside biochemical measure in breast cancer using frozen tissues and mass spectrometry [32]. However, to our knowledge, this is the first report of the NeuGcGM3 expression in FFPE tissues using mass spectrometry.

Previously, Van Cruijsen et al. [9] and Blanco et al. [10] reported that NeuGcGM3 expression is associated with a favorable survival of NSCLC patients. However, in recent studies Hayashi et al. [7] obtained opposite data, considering the reduced number of cases used by Blanco et al. responsible for these differences (*n* = 28). Nevertheless, Van Cruijsen et al. used 165 patients in their research [9], which is a larger sample size compared with the rest of studies [7, 10]. According to our experience, other factors such as the immunohistochemical analysis (membrane and/or cytoplasmic staining), the IHC score, the specificity of Mabs, and the differences in the histological type proportion could be also responsible for these controversial results.

Here, the reactivity of 14F7 Mab in both cell membrane and cytoplasm of malignant cells was analyzed, as it was previously described [10]. However, in previous reports the expression of NeuGcGM3 was only considered in the cytoplasm [9] or in plasmatic membranes [7] of malignant cells, excluding part of Mabs staining. Moreover, the expression of NeuGcGM3 was scored (*F*-score) by mean of the integration of both percentage of 14F7-positive cells and the intensity of reaction. It is known that any scoring decision could directly be influenced by bias because the analysis of IHC assays is visually judged [33]. Nevertheless, the introduction of the score systems in the clinical practice had permitted us to reduce variability, particularly for markers that aim to select patients for specific treatments [34].

Interestingly, NSCLC patients displaying higher levels of NeuGcGM3 expression had a significantly poorer overall survival than those with lower levels of this ganglioside. It is the first report describing the relation between the 14F7 Mab reactivity and a more aggressive biological behavior of NSCLC. Comparable results were recently obtained in colon adenocarcinoma by mean of a similar integrated IHC score [15]. Notably, when only the intensity of reaction or the percentage of 14F7-positive cells was analyzed, in general, no significant associations with clinicopathological features were noted [15], resembling the present research. In this sense, our data permit us to suggest the use of an IHC score integrating both parameters, although further studies are needed to validate these findings.

On the other hand, increasing levels of NeuGcGM3 expression correlated with higher S-Phase fraction and proliferation index measured by flow cytometry. It is known that the upregulation of EGFR expression by EGF is considered a mechanism that promotes the development and progression of lung tumors [35, 36]. Additionally, GM3 diminishes the cancer cell proliferation inhibiting of EGFR tyrosine kinase [37], modulating the expression of cell cycle regulation proteins [38–40]. However, NeuGcGM3 is not able to inhibit the EGFR tyrosine kinase as compared with GM3 [7]. Interestingly, the triple expression of NeuGcGM3 with EGFR and EGF was detected in the 20.0% of studied patients. In line with this, the aberrant expression of NeuGcGM3 in NSCLC probably causes a disruption in the mechanism of EGFR tyrosine kinase inhibition by GM3, permitting an uncontrolled EGFR system activation mediated by the ligand EGF.

In addition, the simultaneous expression of EGFR and its ligands in tumor and adjacent lung tissues was associated with lower overall and relapse-free survival in NSCLC patients [41]. Here, the dual expression of EGFR and EGF was evidenced in 58.7% of NSCLC samples, while the double expression of NeuGcGM3 and EGFR or EGF was detected in 65.0% and 77.5% of these patients, respectively. Outstandingly, patients with EGFR^+^/EGF^+^, NeuGcGM3^+^/EGFR^+^, and NeuGcGM3^+^/EGFR^+^/EGF^+^ phenotypes of NSCLC displayed poor overall survival rates as compared with those with EGFR^+^/EGF^−^, NeuGcGM3^+^/EGFR^−^, and NeuGcGM3^−^/EGFR^+^/EGF^+^, respectively. The dual expression of NeuGcGM3 and EGFR in a variety of human malignant tumors, including NSCLC, was evidenced [42]. However, to our knowledge, it is the first report regarding the double and the triple expressions of NeuGcGM3 with EGFR and/or EGF and its relationship with the overall survival of NSCLC patients.

Finally, differences in predominant histological type might also lead to differences in results. In the first two studies using the 14F7 Mab [9, 10] the proportion of squamous subtype and adenocarcinoma was major in favor of squamous carcinoma, while in the third report an opposite relation was obtained [7], resembling the present work. Lung adenocarcinoma had been reported to be the highest levels of SAT-I mRNA expression as compared with epidermoid carcinoma and the rest of tumors [29]. Interestingly, in this study, a statistically significant difference was obtained when the reactivity of 14F7 Mab was compared in function of the histological subtype of tumors. But no differences in NeuGcGM3 expression were evidenced between epidermoid carcinoma and adenocarcinoma (data not shown). In this sense, experiments using increasing number of these tumors of the lung should be designed.

## 5. Conclusions

In summary, in this paper the expression of NeuGcGM3 in NSCLC is reported for the first time using mass spectrometry and TLC-immunostaining with 14F7 Mab. Interestingly, high levels of NeuGcGM3 expression, by means of an IHC integrated score (*F*-score), correlate with the proliferation index and with a poor overall survival of NSCLC patients. These results support the use of a universal scoring system to assess the prognostic value of NeuGcGM3 expression in NSCLC patients. Moreover, some NSCLC phenotypes with poor overall survival rates based on the simultaneous expression of NeuGcGM3 with EGFR and/or EGF were identified. Our data permit us to consider the immunohistochemical detection of these tumor-associated antigens as a useful complement of established prognostic factors in NSCLC. Moreover, our findings support the potential use of combined passive and active immunotherapy in tumors overexpressing NeuGcGM3, EGFR, and/or EGF.

## Figures and Tables

**Table 1:** Clinicopathological characteristics of NSCLC patients.

Clinicopathological features	Number of cases (%) *n* = 90
Gender	
Female	31 (34.4)
Masculine	59 (65.6)
Age (years)	
<60	58 (64.4)
≥60	32 (35.6)
Tumor size (cm)	
<3	27 (30.0)
≥3	63 (70.0)
Lymph node metastasis	
Yes	30 (33.3)
No	60 (66.7)
Other sites metastasis	
Yes	7 (7.8)
No	83 (92.2)
Stage	
I-II	80 (88.9)
III-IV	10 (11.1)
Disease recurrence	
Yes	32 (35.6)
No	58 (64.4)
Histological type	
Epidermoid carcinoma	26 (28.9)
Adenocarcinoma	43 (47.8)
Large cell carcinoma	8 (8.9)
Carcinoid tumor	9 (10.0)
Others	4 (4.4)
Grade of differentiation	
Well/moderate	55 (61.1)
Poor	35 (38.9)

%, percentage.

**Table 2:** Tissue expression of NeuGcGM3 in NSCLC.

NeuGcGM3 expression	Number of cases (%) *n* = 90
Intensity of reaction	
Negative	6 (6.7)
Weak	15 (16.7)
Moderate	26 (28.9)
Intense	43 (47.8)
Percentage of positive cells (%)	
0–5	6 (6.7)
6–25	9 (10.0)
26–50	17 (18.9)
51–100	58 (64.4)
*F*-score	
Low (<150)	38 (42.2)
High (≥150)	52 (57.8)

%, percentage; *F*-score, final score.

**Table 3:** Expression of NeuGcGM3 in relation to clinicopathological characteristics in NSCLC.

Clinicopathological features (*n* = 90)	NeuGcGM3 expression		*p* value^*∗*^
	(*F*-score)		
	Low	High	
Gender			
Female	15	16	0.469
Masculine	23	36	
Age (years)			
<60	26	32	0.365
≥60	12	20	
Tumor size (cm)			
<3	12	15	0.832
≥3	26	37	
Lymph node metastasis			
Yes	9	21	0.403
No	29	31	
Other sites metastasis			
Yes	3	4	0.669
No	35	48	
Stage			
I-II	35	45	0.562
III-IV	3	7	
Disease recurrence			
Yes	11	21	0.632
No	27	31	
Histological type			
Epidermoid carcinoma	8	18	**0.008** ^*∗∗*^
Adenocarcinoma	15	28	
Others	15	6	
Grade of differentiation			
Well/moderate	22	33	0.905
Poor	16	19	

^*∗*^Fisher exact test; ^*∗∗*^Chi-square test.

**Table 4:** Expression of NeuGcGM3 in relation to ploidy and malignant cells proliferation.

Features	Number of cases (*n* = 51)	NeuGcGM3 expression		*p* value^*∗*^
		Low	High	
Ploidy				
Diploid	25	8	17	1.000
Aneuploid	26	9	17	
S-Phase fraction				
<20%	21	12	9	**0.006**
≥20%	30	5	25	
Proliferation index				
<20%	15	11	4	**0.000**
≥20%	36	6	30	

S-Phase fraction, cells in S-Phase of cell cycle; proliferation index, sum of cells in both S and G_2_/M phases of cell cycle; ^*∗*^Fisher exact test. Bold value indicates statistical significance.

**Table 5:** Univariate and multivariate analyses of overall survival in studied population.

Characteristics	Overall survival		
	Univariate	Multivariate	
	*p* value	*p* value	HR (CI 95%)
Gender	0.309	**0.047**	2.500 (1.014–6.164)
Age	0.739		
Tumor size	0.907	**0.003**	0.162 (0.049–0.528)
Lymph node metastasis	**0.000**		
Other sites metastasis	**0.000**	**0.029**	5.269 (1.190–23.323)
Stage	**0.000**		
Disease recurrence	**0.000**	**0.001**	7.412 (2.311–23.771)
Histological subtype	0.529		
Grade of differentiation	0.952		
NeuGcGM3 expression	**0.020**	**0.010**	3.394 (1.342–8.584)

HR: hazard ratio; CI: confidence interval. Bold value indicates statistical significance.

**Table 6:** Survival of NSCLC patients according to immunohistochemical profile.

Antigens expression	EGFR^+^		NeuGcGM3^+^			
	EGF		EGFR		EGF	
Negative/positive	−	+	−	+	−	+
% of cases	41.3	58.7	51.9	48.1	58.1	41.9
% 5-year survival rate	84.2	48.1	68.2	40.0	61.9	48.0
Median survival (months)	47.5	44.7	44.6	23.3	41.1	38.5
*p* value	**0.014**		**0.030**		0.256	

%, percentage; *p* value, log-rank test. Bold value indicates statistical significance.
